# Multicentric myxoid liposarcoma: report of two cases

**DOI:** 10.1186/1477-7819-5-139

**Published:** 2007-12-10

**Authors:** M Jesus Fernández-Aceñero, Pilar López-Criado, Mariano López-Franco, Telma Meizoso, Carolina Calvo

**Affiliations:** 1 Oncology, Hospital General of Móstoles, Madrid, Spain; 2 Orthopaedic Surgery, Hospital General of Móstoles, Madrid, Spain; 3 Radiology. Hospital General of Móstoles, Madrid, Spain; 4 Department of Surgical Pathology, Hospital General of Móstoles, Madrid, Spain

## Abstract

**Background:**

Multicentric myxoid liposarcoma is a rather infrequent tumour that tends to behave aggressively.

**Case presentation:**

We herein report two further cases of this tumour that have been managed in our Hospital. Both were young men with multiple sites of involvement at the moment of diagnosis and both have shown a bad prognosis with frequent recurrences after treatment and rapid death in one case.

**Conclusion:**

We comment on the diagnosis of this entity and on the therapeutic options available for these patients.

## Background

Liposarcomas are among the most frequent malignant soft tissue tumours. Most of them arise in the extremities or the retroperitoneum, affect middle-aged and old patients and tend to follow a relatively indolent clinical course with local recurrences after resection and occasional distant metastasis, mainly to the lungs [1]. Prognosis is guarded, especially for deep-seated lesions, but long-term survival is described, mainly for the best-differentiated histological types, including well-differentiated lipoma-like liposarcoma, that comprise 40%–50% of these tumours. Myxoid liposarcoma is a distinct histological type of liposarcoma with an intermediate prognosis between well-differentiated tumours and pleomorphic ones. However, some of the myxoid liposarcomas, mainly those arising in the extremities, are multicentric in nature, tend to affect younger patients and follow a rather aggressive clinical course, leading to death [2]. In the present work we report two further cases of this rare clinical entity and comment on diagnostic and management issues.

## Case presentation

### Case 1

**Figure 1:**
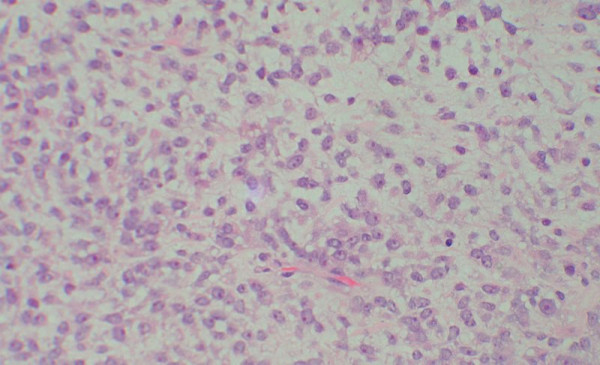
myxoid tumour with some lipoblasts (H&E × 400).

**Figure 2:**
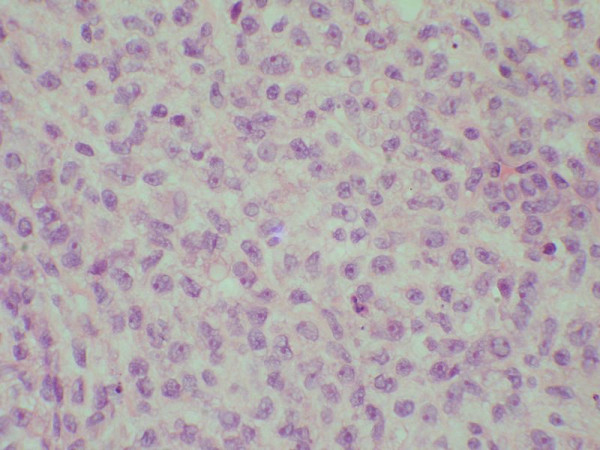
round cell areas in the tumour (H&E × 400).

A 44-year-old man consulted on pain affecting his left leg. His previous medical record was uneventful with no previous hospital admissions or surgical interventions. The patient reported no weight loss and had no other symptoms rather than pain, which had progressively worsened in the last 2 months and prevented the patient from sleeping at night. The physical examination was unremarkable, but the MRI performed showed a huge soft tissue mass, affecting the adductor muscles of the left thigh and measuring 30 × 20 cm. Radiological diagnosis were liposarcoma or haemangioma. An incisional biopsy of the mass revealed a diffuse growing neoplasm. The predominant areas were of myxoid nature (Figure 1), but there were also foci of round less-differentiated cells (Figure 2). Immunohistochemistry showed a positive reaction for S100 protein, while the other markers (for muscle, vascular and epithelial differentiation and also p53 protein) were negative. The extension imaging study including CAT showed no evidence of metastasis and with the histopathological diagnosis of myxoid/round cell liposarcoma the patient underwent amputation of his left leg at the hip level, 15 days after the incisional biopsy. During the postoperative period of the first surgery he suddenly developed intense pain in his right leg, which he had not reported before. Physical examination revealed a 7 cm soft mass in the right hamstring and a core needle biopsy showed a tumour with the same histopathological features as those found in the left leg. The patient refused surgery and he underwent local radiotherapy of the mass. One year after initial diagnosis the routine follow-up CAT revealed a huge retroperitoneal mass suggestive of adipose origin. Chemotherapy was initiated with iphosphamide, adriamycin and DTIC with apparent stabilization of the mass. The general situation of the patient gradually worsened and he died with persistence of the retroperitoneal disease 23 months after initial diagnosis. No autopsy was allowed.

### Case 2

**Figure 3:**
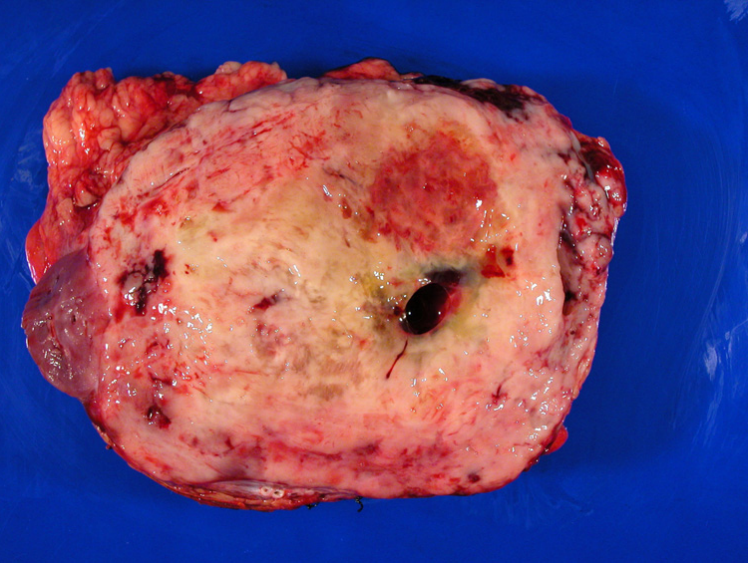
recurrence of the adipose mass with kidney involvement.

A 35-year-old man consulted on the appearance of a right gluteal mass. He had noticed the mass some months before, but he was not concerned about it for he had considered it the result of the intensive training due to his job as a bodyguard. However, the mass had grown so much that he could no longer sit and he decided to seek medical assistance. The patient was in good general health, had no other symptoms and reported no weight loss. The physical examination confirmed a 40 × 15 cm mass in his right gluteal area and the MRI suggested a possible adipose nature for it. Incisional biopsy showed a similar histological image to that described in the previous case with myxoid and round cell areas. Diagnosis was myxoid liposarcoma and material from the biopsy was sent for cytogenetic analysis and subsequently confirmed the translocation t(12;16) (q13;p11) typical of myxoid and round cell liposarcomas. During the extension study the CAT showed a 7-cm extrapulmonary mass in the posterior mediastinum. The core needle biopsy showed the same histological image as the gluteal mass. As surgical therapy would imply amputation of the leg including the hip and the pelvis (hemipelvectomy) and the patient had a second mass in the mediastinum, neodajuvant chemotherapy was initiated with iphosphamide, adriamycin and DTIC. The control MRI indicated partial response of the mass to chemotherapy and two months after diagnosis simple resection of the gluteal mass was attempted. The histological analysis of the enucleated mass showed less than 10% of tumour necrosis related to chemotherapy and confirmed the existence of widespread areas of round cells, comprising almost 40% of the tumour. After surgery local radiotherapy was initiated with good tolerance. For one year the patient remained well and apparently disease-free, but 13 months after initial diagnosis he consulted on the appearance of a right laterocervical mass. Fine-needle aspiration suggested an adipose tumour and a cervical lymphadenectomy confirmed involvement by myxoid liposarcoma in one of twelve identified lymph nodes, without any evidence of disease elsewhere. No further therapy was initiated and the patient remained well, but 8 months after this last surgery he suddenly developed abdominal pain and the imaging techniques revealed a 18 cm adipose mass involving the left kidney. The patient was operated again for bulk reduction and underwent wide resection of the retroperitoneal mass including the left adrenal gland and kidney (Figure 3). However, macroscopic residual disease persisted. Today, 25 months after initial diagnosis he remains alive with progressive disease and is under palliative care.

## Discussion

Liposarcomas are the most frequent soft-tissue sarcomas. The World Health Organization Classification of Tumours [1] divides these tumours in four main histological types: well-differentiated, myxoid, round cell and pleomorphic types. As myxoid and round cell tumours share the same cytogenetic abnormalities, namely the traslocation t(12;16)(q13;p11) leading to the fusion of the genes *DDIT3 *and *FUS *with generation of a hybrid protein FUS/DDIT3, some authors consider both lesions as a continuum of the same disease. This possibility seems to be supported by the frequent finding of areas of round cells in myxoid liposarcomas, which has been considered a marker of poor prognosis when representing 5% or more of the mass in localized myxoid liposarcoma. In our second patient the cytogenetic analysis performed in paraffin-embedded tissue from the tumour confirmed the typical traslocation; in the first patient the specimen was not suited for cytogenetic analysis.

In the present work we report two patients with a multicentric myxoid liposarcoma. This entity seems to be rather infrequent and some authors consider it different from ordinary liposarcoma due both to multicentric presentation and to a more aggressive behaviour [2,3]. There has been some debate whether these multicentric tumours truly represent synchronous lesions at different levels or a haematogenous spread from one primary lesion [4]. Interestingly the multicentric tumours tend to spare classical metastatic sites of sarcomas, like the liver, the lungs or the bone and affect rare locations, like the pleura or the lymph nodes, as happened in one of our patients [5]. This fact seems to speak against a possible metastatic explanation for multicentricity. The identification of the same cytogenetic abnormalities in all the multicentric lesions cannot be considered either definite proof of their metastatic origin, for they might still be multicentric synchronous or metachronous lesions related to a common aetiopathogenic factor [6].

Another important fact highlighted by our cases is the importance of performing a complete imaging study of patients with myxoid liposarcomas of the limbs, mainly to exclude possible multicentric lesions [7].

As for therapy it seems surgery remains as the mainstay of treatment [8]. In our patient the initial surgical management included limb amputation before diagnosis of multicentrity was performed. This form of therapy might be considered rather too aggressive; if we had known that the patient had more lesions, we would have applied neoadjuvant chemotherapy for bulk reduction and perform a more conservative surgical approach after it, like we did in the second case. However, at the time of the first surgery we had no sign of multicentric involvement by the tumour and we chose the possible curative alternative for a young person in a good general health. Nevertheless, bulk reduction followed by surgery has not seemed to change the fate in our second case, for the tumour has shown the same tendency to recur and has behaved as aggressively as in the first case.

As prognosis seems to be poor, chemotherapy and radiotherapy seem indicated both in the neoadjuvant or adjuvant settings. As these multicentric tumours are rare, it is difficult to determine which regimen could be the best in these patients. Nevertheless, the literature does not indicate a significant improvement of the outcome regardless of the chosen therapy.

## Conclusion

The management of multicentric myxoid-round cell liposarcomas seems far from settled and prognosis seems poor despite different therapeutic options. We report two further cases, both with a bad prognosis despite aggressive therapy, confirming the general impression found in the world literature regarding this entity.

## Competing interests

The author(s) declare that they have no competing interests.

## Authors' contributions

**MJFA **diagnosed the first case, reviewed the literature on this issue and written the clinical report; **PLC **was the oncologist in charge of both patients and has written the therapy section; **MLF **operated both patients and helped in preparation of draft manuscript; **TM **diagnosed histologically the second case and helped with pathological section of manuscript; and **CC **established the radiological diagnosis in both cases and helped with radiological section of manuscript. All authors read and approved final manuscript
